# In Memory of Academician Er-Mi Zhao (1930-2016)

**DOI:** 10.13918/j.issn.2095-8137.2017.006

**Published:** 2017-01-18


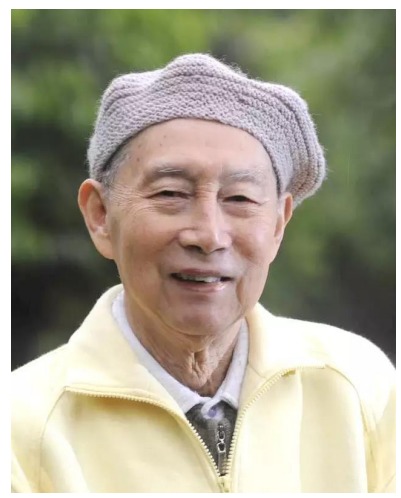


**Professor Er-Mi Zhao, Herpetologist, 1930-2016**

Academician Er-Mi Zhao, Professor of the Chengdu Institute of Biology, Chinese Academy of Sciences, and Sichuan University, passed away on 24 December, 2016, to the great loss of both Chinese and world herpetology. As one of the most internationally renowned Chinese herpetologists, Academician Zhao represented a remarkable era of Chinese herpetology and made a significant contribution to global research, both academically and spiritually. He played a significant role in our understanding of the amphibians and reptiles of Tibet, herpetofauna of the Hengduan Mountains, taxonomy of Chinese snakes, and biogeography of the East Asian islands. His distinguished studies laid a solid and substantial foundation for research by future generations of young herpetologists, and had an extensive and profound impact on the development of the field.

As a prestigious scientist, Academician Zhao won the recognition and respect of his international peers and assured his place on the worldwide stage based on his remarkable academic achievements. "China should be so proud to have you representing her in herpetology research", Roger Conant, a senior American scientist and *Agkistrodon* expert, wrote to Academician Zhao in 1989. In 1993, *Herpetology of China* (Zhao & Adler, 1993), the first book on the herpetofauna of China, which systematically described the 661 known species at the time, was co-authored by Academician Zhao and American scholar Prof. Kraig Adler. This great work took nearly half a century of meticulous investigation and ten years of compilation to achieve. The book was highly praised by Academician Ilya Darevsky of the Russian Academy of Sciences and Academician David Wake of the American Academy of Sciences, who claimed it to be "a milestone of herpetology literature" and "an epoch-making book, [which] inaugurates a new era in exploring the fauna of amphibians and reptiles of this vast land". 

As a diligent researcher, Academician Zhao devoted himself to herpetology research for 60 years. During his academic career, he published more than 140 articles, 43 books, including *Fauna Sinica* (Zhao et al., 1998), *Snakes of China* (Zhao, 2006), *Atlas of Chinese Snake*s (Hu et al., 1980), *Amphibian Zoogeographic Division of China* (Zhao, 1995), and *Herpetology of China* (Zhao & Adler, 1993) in five translations, and established four periodicals. Many of his works were compulsory study for herpetologists and students in China, America, England, and various other countries.

For herpetological classification and zoogeographic regionalization, Academician Zhao discovered a new record family of snake, *Cylindrophis ruffus* (Zhao & Adler, 1989), and 17 new record species of amphibians and reptiles in China. In addition, two new genera of amphibians, *Satobius* (*Hynobius retardatus*) (Adler & Zhao 1990) and *Liua* (*Liua shihi*) (Zhao & Hu, 1983), and 41 new species (or subspecies) were described and named by him, including *Japalura szechwanensis* (Hu & Zhao 1966), *Achalinus meiguensis* (Hu & Zhao, 1966), *Natrix optata* (Hu & Zhao, 1966). *Ranodon tsinpaensis* (Hu et al., 1966), *Megophrys nankiangensis* (Hu et al., 1966), *Hyla tsinlingensis* (Hu et al., 1966), *Rana kuangwuensis* (Hu et al., 1966), *Microhyla mixtura* (Hu et al., 1966), *Leiolopisma tsinlingensis* (Hu et al., 1966), *Xenopeltis hainanensis* (Hu et al., 1975), *Dinodon rosozonatum* (Hu et al., 1975), *Achalinus hainanus* (Hu et al., 1975), *Trimeresurus medoensis* (Zhao & Jiang, 1977), *Ovophis monticola zayuensis* (Zhao & Jiang, 1977), *Plagiopholis unipostocularis* (Zhao et al., 1978), *Opisthotropis guangxiensis* (Zhao et al., 1978), *Agkistrodon shedaoensis* (Zhao, 1979a), *Trimeresurus xiangchengensis* (Zhao, 1979b), *Phrynocephalus vlangalii hongyuanensis* (Jiang et al., 1980), *Oligodon multizonatum* (Zhao & Jiang, 1981), *Macropisthodon rudis multiprefrontalis* (Zhao & Jiang, 1981), *Scincella huanrenensis* (Zhao & Huang, 1982), *Scutiger ruginosus* (Zhao & Jiang, 1982), *Rhabdophis nuchalis pentasupralabialis* (Jiang & Zhao, 1983), *Platymantis reticulatus* (Zhao & Li, 1984a), *Staurois liangshanensis* (Wu & Zhao, 1984), *Calotes medogensis* (Zhao & Li, 1984b), *Tenuidactylus medogensis* (Zhao & Li, 1987), *Rana tenggerensis* (Zhao et al., 1988), *Eumeces liui* (Hikida & Zhao, 1989), *Trimeresurus mangshanensis* (Zhao & Chen, 1990), *Cuora zhoui* (Zhao et al, 1990), *Amphiesma metusia* (Inger et al, 1990), *Oreolalax multipunctatus* (Wu et al., 1993), *Rana robertingeri* (Wu & Zhao, 1995), *Rhabdophis adleri* (Zhao, 1997), *Laudakia wui* (Zhao, 1998a), *Laudakia papenfussi* (Zhao, 1998b), *Rana zhengi* (Zhao, 1999), *Opisthotropis cheni* (Zhao, 1999), *Rhacophorus hainanus* (Zhao et al., 2005), and *Gloydius lijianlii* (Jiang & Zhao, 2009), as well as the world-renowned Shedao Island pit viper (*Gloydius shedaoensis*) (Zhao, 1979a), Medog green pit viper (*Viridovipera medoensis*) (Zhao & Jiang, 1977), and Mt. Mang pit viper (*Protobothrops mangshanensis*) (Zhao & Chen, 1990). He also added eight new species for Tibet and 10 newly recorded species in China, and was the first to report on the king cobra in Medog (Zhao & Li, 1983) and expand its distribution northwards (Zhao et al., 1986).

Academician Zhao brought new insight and perspective on the geographical division of amphibians and reptiles in China, the amphibian and reptile fauna in the Hengduan Mountain area, the classification of snakes distributed in China, and the zoological geography of the East Asian Islands. He zoned the southern slopes of the Himalayas as a new sub-southwest region of the Indo-Chinese sub-region in the oriental realm according to the actual distribution of species in Tibet, and first proposed a new "South of the Himalayan Sub-region" based on the distribution of reptiles (Zhao et al., 1986). In addition, Academician Zhao also proposed new distribution patterns of amphibians in extra-tropical East Asia and the formation of amphibians and reptiles in the Western Pacific island chain (Zhao, 1989).

Professor Zhao also did outstanding work in scientific practice. He developed measures for the prevention and management of venomous snakes in the grasslands in western Xinjiang, and advanced the concept of medical geography of venomous snake bites in China (Qian et al., 1991) and Yunnan snake medicine to guide the practice of snake bite control. Collaborating with colleges and universities in Hainan, Professor Zhao recruited Masters and Doctoral aquaculture students to cultivate professional personnel for python breeding. He actively cooperated with Hainan python farms by providing systematic scientific knowledge.

Academician Zhao was not only a devoted scholar, but also played active roles in helping young researchers and in disseminating scientific knowledge. He served on the Executive Committee of the International Amphibian and Reptilian Society in 1983, and as the Chairman of Chinese Herpetologists in the IUCN (1991-2001). During his tenure as an academic advisor on the Editorial Board of *Zoological Research* from 1997 to 2014, he fulfilled his duty faithfully and diligently, and provided vital and constructive promotion of the journal. *Zoological Research* is extremely grateful for his exceptional contribution and dedication, and it is with deep regret and sorrow we note his passing. His deep passion, persistence, optimism, and strict scientific attitude in chasing the truth showed his invaluable spiritual wealth, and is an inspiration to every one of us.

**Editor's note: **The facts and material stated here in regards to Academician Zhao's academic achievements were provided by his students, Dr. Jia-Tang Li (Chengdu Institute of Biology, CAS), Dr. Jing Che (Kunming Institute of Zoology, CAS), Dr. Song Huang (Huangshan University, China), Peng Guo (Yibin University, China) and Dr. Yue-Zhao Wang (Chengdu Institute of Biology, CAS). This essay was compiled by *Zoological Research*.

## References

[1] Adler KA, Zhao EM. 1990 Studies on hynobiid salamanders, with description of a new genus. * Asiatic Herpetological Research*, 3 37- 45.

[2] Hikida T, Zhao EM. 1989 * Eumeces liui*:a new species of blue-tailed skink (Lacertilia:Scincidae) from China. * Copeia*, 1989 (1): 110- 114.

[3] Hu BQ, Huang MH, Xie ZT, Zhao EM, Jiang YM, Huang QY, Zong Y, Ma JF. 1980 . * Atlas of Chinese Snakes*, Shanghai Shanghai Scientific and Technical Publishers (in Chinese)

[4] Hu SQ, Zhao EM, Liu CZ. 1966 A herpetological survey of the Tsinling and Ta-Pa Shan Region. * Acta Zoologica Sinica*, 18 (1): 57- 92. (in Chinese)

[5] Hu SQ, Zhao EM. 1966 Three new species of reptiles from Szechwan. * Acta Zootaxonomica Sinica*, 3 (2): 158- 164. (in Chinese)

[6] Sichuan Biological Research Institute, Beijing Institute of Zoology. 1975 Three new species of reptiles from Hainan Island, Guangdong Province. * Acta Zoologica Sinica*, 21 (4): 379- 384.

[7] Inger RF, Zhao EM, Shaffer BH, Wu GF. 1990 . * Report on A Collection of Amphibians and Reptiles from Sichuan, China*, Chicago Field Museum of Natural (in Chinese)

[8] Jiang F, Zhao EM. 2009 * Gloydius lijianlii*, a new species from the Northern Coastal Islands along Shandong Peninsula (Reptilia, Squamata, Viperidae). * Acta Zootaxonomica Sinica*, 34 (3): 642- 646. (in Chinese)

[9] Jiang YM, Huang QY, Zhao EM. 1980 A new subspecies of* Phrynocephalus vlangalii* (Strauch) and preliminary observations on its ecology. * Acta Zoologica Sinica*, 26 (2): 178- 183. (in Chinese)

[10] Jiang YM, Zhao EM. 1983 Studies on amphibians and reptiles of Mt. Gongga region, Sichuan, China. 3. A study of speciesgroup nuchalis, genus* Rhabdophis*.* Acta Herpetologica Sinica*, 2 (1): 59- 62. (in Chinese)

[11] Qian Y, Zhao EM, Zhao KT. 1991 Animal Science Research:A Symposium Issued to Celebrate the 90th Birthday of the Professor Mangven Ly Chang, Beijing Chinese Forestry Press :, 208- 216. (in Chinese)

[12] Wu GF, Zhao EM. 1984 A rare karyotype of anurans, the karyotype of* Rana phrynoides*. * Acta Herpetologica Sinica*, 3 (4): 5- 10. (in Chinese)

[13] Wu GF, Zhao EM, Inger RF, Shaffer HB. 1993 A new frog of the genus* Oreolalax* (Pelobatidae) from Sichuan, China. * Journal of Herpetology*, 27 (4): 410- 413.

[14] Wu GF, Zhao EM. 1995 A new ranid species of the spinosae group from Sichuan.* In*:Zhao EM. Amphibian Zoogeographic Division of China. A Symposium Issued to Celebrate the Second Asian Herpetological Meeting Held at Ashgabat, Turkmenistan 6 to 10 September 1995. Sichuan Journal of Zoology, Supplement, 52- 54. Chengdu, China. (in Chinese)

[15] Herpetological Department, Sichuan Biological Research Institute. 1977 A survey of reptiles in Xizang Autonomous Region, with faunal analysis and descriptions of new forms. * Acta Zoologica** Sinica*, 23 (1): 64- 71. (in Chinese)

[16] Zhao EM, Jiang YM, Huang QY. 1978 Three new Colubrid species in China. * Materials for Herpetological Research*, 4 21- (in Chinese)

[17] Zhao EM. 1979a. A new Agkistrodon from Shedao (Snake Island), Liaoning. * Acta Herpetologica Sinica*, 1 (1): 4- 6. (in Chinese)

[18] Zhao EM. 1979b. A new snake of the genus* Trimeresurus* from Sichuan, China. * Acta Zootaxonomica Sinica*, 4 (4): 422- 424. (in Chinese)

[19] Zhao EM, Jiang YM. 1981 Studies on amphibians and reptiles of Mt. Gongga Shan, Sichuan, China, I. A new species and a new subspecies of snakes from Sichuan. * Acta Herpetologica Sinica*, 5 (7): 53- 58. (in Chinese)

[20] Zhao EM, Huang KC. 1982 A survey of amphibians and reptiles in Liaoning Province. * Acta Herpetologica Sinica*, 1 (1): 1- 23. (in Chinese)

[21] Zhao EM, Jiang YM. 1982 Studies on amphibians and reptiles of Mt. Gongga Shan, Sichuan, China. 2. A new species of genus* Scutiger* (Amphibia:Pelobatidae). * Acta Herpetologica Sinica*, 1 (1): 79- 82. (in Chinese)

[22] Zhao EM, Hu QX. 1983 Taxonomy and evolution of Hynobiidae in western China, with description of a new genus. * Acta Herpetologica Sinica*, 2 (2): 29- 35. (in Chinese)

[23] Zhao EM, Li SQ. 1983 * Ophiophagus hannah* (Cantor), a Record New to Xizang (Tibet) Autonomous Region, China. * Acta Herpetologica Sinica*, 2 (4): 44- (in Chinese)

[24] Zhao EM, Li SQ. 1984a. A new species of the genus* Platymantis* (Amphibia:Ranidae) from Xizang. * Acta Herpetologica Sinica*, 3 (3): 55- 57. (in Chinese)

[25] Zhao EM, Li SQ. 1984b. A new species of* Calotes* (Lacertilia:Agamidae) from Xizang (Tibet). * Acta Herpetologica Sinica*, 3 (4): 77- 78. (in Chinese)

[26] Zhao EM, Jiang YM, Li SQ. 1986 Reptilian faunal analysis and zoogeographical division of Xizang Autonomous Region. * Acta Herpetologica** Sinica*, 5 (3): 199- 203. (in Chinese)

[27] Zhao EM, Li SQ. 1987 A new lizard of* Tenuidactylus* and a new Tibetan snake record of Amphiesma. * Acta Herpetologica Sinica*, 6 (1): 48- 51. (in Chinese)

[28] Zhao EM, Macey JR, Papenfuss TJ. 1988 A new species of Rana from Ningxia Hui Autonomous Region. * Chinese Herpetological Research*, 2 (1): 1- 3.

[29] Zhao EM, Adler K. 1989 Asian pipe snake——A snake family new to China. * Sichuan Journal of Zoology*, 8 (2): 26- (in Chinese)

[30] Zhao EM. 1989 Biogeography of the Amphibians and Reptiles of the Main Islands of East Asia. London:Q. E. D. Recording Services Ltd. Tape 7,

[31] Zhao EM, Chen YH. 1990 Description of a new species of the genus* Trimeresurus*. * Sichuan Journal of Zoology*, 9 (1): 11- 12. (in Chinese)

[32] Zhao EM, Zhou T, Ye P. 1990 A new Chinese box turtle (Testudinata:Emydidae)-*Cuora zhoui*.* In*:Zhao EM (editor). From Water onto Land. A symposium issued to commemorate the ninetieth birthday of Professor Cheng-Chao Liu, a distinguished Herpetologist of China. Beijing:Chinese Forestry Press, 213- 216. (in Chinese)

[33] Zhao EM, Adler K. 1993 Herpetology of China. Oxford, Ohio Society for the study of Amphibians & Reptiles

[34] Zhao EM. 1995 Amphibian Zoogeographic Division of China. A Symposium Issued to Celebrate the Second Asian Herpetological Meeting Held at Ashgabat, Turkmenistan 6 to 10 September 1995. Sichuan Journal of Zoology, Supplement, 52- 54. Chengdu, China. (in Chinese)

[35] Zhao EM. 1997 A new species of* Rhabdophis* (Serpentes:Colubridae) from Hainan Island, China. * Asiatic Herpetological Research*, 7 166- 169.

[36] Zhao EM, Huang MH, Zong Yu . * Fauna Sinica*:Reptilia, Vol. 3, Squamata, Serpentes, Beijing Science Press 1998 (in Chinese)

[37] Zhao EM. 1998a. A new species of the genus* Laudakia* from Xizang (Tibet) Autonomous Region (Sauria:Agamidae). * Acta Zootaxonomica Sinica*, 23 (4): 440- 444.

[38] Zhao EM. 1998b. Description of a new species of the genus* Laudakia* from Xizang (Tibet) (Sauria:Agamidae). * Zoological Research*, 19 (5): 401- 404.

[39] Zhao EM. 1999 Diagnoses of a new frog and a new snake from China. * Sichuan Journal of Zoology*, 18 (3): cover 2- (in Chinese)

[40] Zhao EM, Wang LJ, Shi HT, Wu GF, Zhao H. 2005 Chinese Rhacophorid frogs and description of a new Species of* Rhacophorus*. * Sichuan Journal of Zoology*, 24 (3): 297- 300. (in Chinese)

[41] Zhao EM. 2006 . Snakes of China, Hefei Anhui Science and Technology Publishing House (in Chinese)

